# A Novel Target Detection Method of the Unmanned Surface Vehicle under All-Weather Conditions with an Improved YOLOV3

**DOI:** 10.3390/s20174885

**Published:** 2020-08-28

**Authors:** Yan Li, Jiahong Guo, Xiaomin Guo, Kaizhou Liu, Wentao Zhao, Yeteng Luo, Zhenyu Wang

**Affiliations:** 1 The State Key Laboratory of Robotics, Shenyang Institute of Automation, Chinese Academy of Sciences, Shenyang 110016, China; guojiahong@sia.cn (J.G.); guoxiaomin@sia.cn (X.G.); Liukzh@sia.cn (K.L.); zhaowt@sia.cn (W.Z.); luoyeteng@sia.cn (Y.L.); wangzhenyu@sia.cn (Z.W.); 2 Institutes of Robotics and Intelligent Manufacturing, Chinese Academy of Sciences, Shenyang 110169, China; 3 University of Chinese Academy of Sciences, Beijing 100049, China; 4 School of Automation and Electrical Engineering, Shenyang Ligong University, Shenyang 110159, China; 5 Shenyang Institute of Automation, Guangzhou, Chinese Academy of Sciences, Guangzhou 511458, China

**Keywords:** unmanned surface vehicle, real-time object detection, deep learning, YOLOV3, all-weather condition

## Abstract

The USV (unmanned surface vehicle) is playing an important role in many tasks such as marine environmental observation and maritime security, for the advantages of high autonomy and mobility. Detecting the targets on the surface of the water with high precision ensures the subsequent task implementation. However, the changes from the lights and the surface environment influence the performance of the target detecting method in a long-term task with USV. Therefore, this paper proposed a novel target detection method by fusing DenseNet in YOLOV3 to improve the stability of detection to decrease the feature loss, while the target feature is transmitted in the layers of a deep neural network. All the image data used to train and test the proposed method were obtained in the real ocean environment with a USV in the South China Sea during a one month sea trial in November 2019. The experiment results demonstrate the performance of the proposed method is more suitable for the changed weather conditions though comparing with the existing methods, and the real-time performance is available in practical ocean tasks for USV.

## 1. Introduction

In recent years, the unmanned surface vehicle (USV) as a typical automatic unmanned system has made considerable and rapid development. It is playing an important role in both military and civilian missions to reduce human casualties as well as to create mission efficiencies, covering submarine tracking, environmental monitoring, patrol, reconnaissance, and so on [1]. Furthermore, USVs are also implemented in hydrographic measurement or bathymetric survey in shallow water regions because of some of their special advantages [2,3,4,5]. The autonomous and reliable navigation without obstacle collision is one of the important preconditions to ensure the completion of these tasks. To achieve superior perception performance, the USV generally requires employing heterogeneous sensors covering radar, lidar, camera, and infrared sensors [6]. They provide advantages of computer vision in terms of power consumption, size, weight, cost, and the readability of data, unlike radar or LIDAR, which may require heavy equipment placed on the vehicle [7,8,9]. Therefore, vision-based target detection on the sea for USVs has received much attention. Meanwhile, the camera is becoming one of the necessary equipments for USV to perform environment perception and object detection, especially for small and low-end vehicles, and the development of highly efficient computer vision algorithms for object detection is a huge challenge in the real complex environment [10].

In previous literatures, many researchers have contributed and provided different methods to detect objects on the water surface. The traditional methods usually detect the horizon line to distinguish the water region and the sky/land region in advance, and then several ad hoc image processing algorithms are implemented to segment the potential objects in the water region below the horizon [11,12,13,14]. Although these methods are suited for the undisturbed water surface, it is difficult to search the horizon line under a complex dynamic environment (haze, fog, sn glitter, and so on) or in the areas closed to the shoreline or in a marina. These limit the applications of USVs in open water fields. In these works, manual set low-level features such as the color [14] are used to recognize the objects, which could tend to cause false detection influenced by the real environment [15].

With the development of machine learning, deep learning algorithms have been widely used in object detection. In particular, as the typical representative of deep learning methods, the convolutional neural network (CNN) has been successfully employed in many applications. Owing to the advantages in high-level feature extraction and representation from raw data automatically, CNN has achieved more significant performance in image classification [16,17,18] and speech recognition [19,20]. Faster R-CNN, proposed in 2015 [21] as an improved form of CNN with region proposal networks, has been implemented in object detection for USVs in recent years, and the identification accuracy is improved compared with other methods [22,23,24].

However, the complex realistic external environment brings large challenges to the performance of object detection. It is regrettable that, in these works, no environmental factors are taken into account in object detection. While in the long-term task, for instance, changes of light, water droplets adhere to the lens of the camera, changes in vehicle attitude caused by current, even sea fog, and water reflection take place, all environmental changes leading to the instability of the original detection method [6,25].

YOLO (You only look once) [26,27,28] is different from the region-based deep learning methods such as R-CNN (region-based convolutional neural network) [29]. A YOLO network directly performs regression to detect targets in images without requiring the region proposal network (RPN) to detect regions of interest in advance, which speeds up the detection of the target processes [30]. As the state-of-the-art version, YOLOV3 could detect the targets with high accuracy and speed, and also performs well in the detection of small-size targets. These advantages ensure that the USV could detect the targets in real time even if the targets are still far from the USV. Thus, we proposed the object detection method for USV based on YOLOV3 in this paper. Simultaneously, DenseNet is employed to improve the original YOLOV3, which could increase the stability of the detection method to the changes of the dynamic environment by reducing the feature loss in the object detection process.

The main contributions of this paper are summarized as follows. (1) Although the YOLOV3 model has an excellent performance in small-size target detection, the ever-changing dynamic ocean environments suppress the detection performance for the reason that partial features of the target are lost during the features transmitting in the deep neural network owing to both convolution and down-sampling operation. The proposed YOLOV3-dense model ensures all the features transmit with no loss between layers of deep neural network and improves the reusability of features. (2) The raw images are fed to the deep neural network as a training dataset, and the features of the raw images cannot characterize all-weather conditions and sea condition changes owing to the constraints of data acquisition platform and sea trial period, which also limist the capability of detection under the all-weather conditions. In this paper, the training dataset is augmented to rich features of weather conditions by adjusting the brightness and rotation, which improves the robustness of the model to changes in environmental conditions.

The rest of this paper is organized as follows. In Section 2, we introduce the image data pre-processing, including data acquisition and augmentation. Next, the target detection method with an improved YOLOV3 is proposed in Section 3. The evaluation of target detection in some factors is addressed in Section 4, while the experimental results are discussed in Section 5. Finally, the conclusions are provided in Section 6.

## 2. Image Data Pre-Processing

### 2.1. Image Data Acquistion

**Figure 1:**
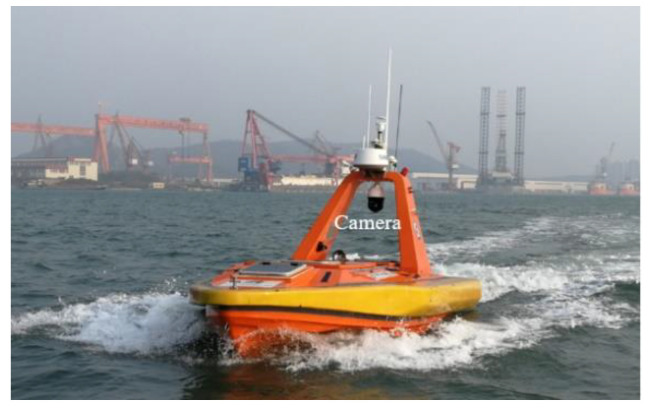
Unmanned surface vehicle (USV) platform and visual system installation.

In this study, image acquisition was conducted using a forward-looking camera with 1280 × 720 pixel resolution. The camera was installed horizontally on the top of the USV developed by Shenyang Institute of Automation, Chinese Academy of Sciences to monitor the surface of the water. The USV platform and the visual system installation are shown in Figure 1. The camera model is the iDS-2DF8837I5X of Hikvision with 8 megapixels.

The image data used in this paper were collected in the South China Sea during a one month sea trial in November 2019 under sunny weather and cloudy conditions. The collection periods were throughout the day from 07:00 to 17:00. The ship was selected as the target in this paper and 3000 images of ships were collected. The collected image data covered as many different environmental conditions as possible during the day. The environmental conditions and basic parameters of the collected images are shown in Table 1. A total of 1000 ship images were randomly selected for use as the training dataset.

To make the collected image data reflect more different environmental conditions, the 1000 images were then expanded to 4000 images by implementing data augmentation methods to yield the training dataset.

### 2.2. Image Data Augmentation

**Figure 2:**
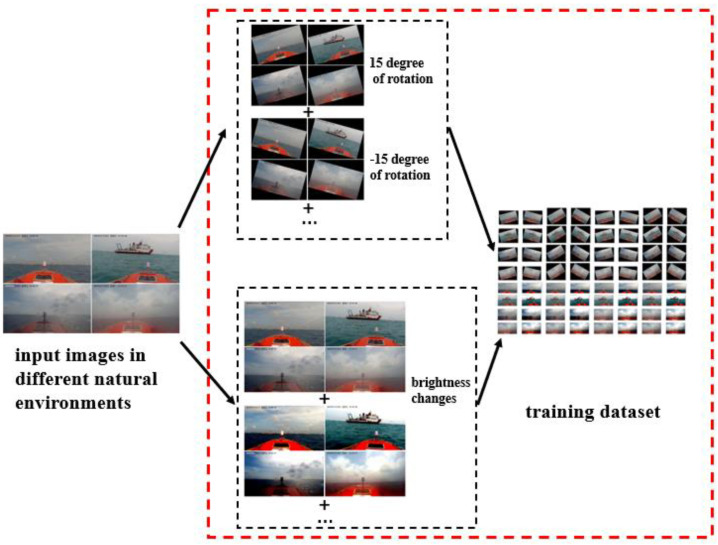
Framework of data augmentation.

Considering that the intensity of light illumination and USV attitude caused by the waves vary greatly during the day, this could influence the performance in both the model training and method verifying steps. Therefore, the dataset used to train the model was augmented to enrich the experimental dataset by adjusting the brightness and rotation. This step of training dataset augmentation not only should enrich the deep feature maps of targets, but also could be considered to improve the robustness of the target detection method in the realistic environment condition. The framework of data augmentation is shown in Figure 2.

#### 2.2.1. Data Augmentation: Brightness

To improve the robustness to the luminance varies of the natural light, the original images were augmented by adjusting the brightness, and the pre-processed images were added to the training dataset. The threshold values were randomly set in the range between $l_{min}$ and $l_{max}$. However, if the brightness images are set too high or too low, the image annotation will become difficult because the edge of the target is unclear for manual annotation. On the other hand, these images will also influence the performance of the model training. Therefore, there will be some constraints in the threshold values selection; in this work, the threshold values of the brightness were set to [0.3, 0.7] for $l_{min}$ and $l_{max}$, respectively.

#### 2.2.2. Data Augmentation: Rotation

Considering the influence on camera attitude from the sea waves, especially USV sailing at high speed, the training dataset was also manual augmented by rotating the image data with different angular degrees. Here, 15° and −15° were utilized in our work, thus the training data were enhanced three times after rotation. The rotated images can also improve the detection performance of the proposed method.

### 2.3. Image Annotation

To compare the performance with other algorithms, the images for training the model weights were converted to PASCAL VOC format. The targets in training images were labeled manually by drawing bounding boxes with a software called LabelImg, which is a graphical annotation tool designed for use in deep learning algorithms. The completed dataset is shown in Table 2.

## 3. Methodologies

### 3.1. YOLOV3

YOLO was proposed by Redmon et al. in 2016 [26], and the core structure of YOLO is a convolutional neural network that can predict multi-class targets at one time. It can realize the end-to-end target detection in a real sense, with advantages in terms of high detection accurate rate and fast speed. YOLOV3 was released in 2018, and is the state-of-the-art version of YOLO [27].

YOLO divides the input image into a grid. If the center point of the object’s ground truth falls within a certain grid, the grid is responsible for detecting the object. Each grid outputs prediction bounding boxes, and the information for each bounding box contains five values (x,y,width,height, and prediction confidence). The prediction confidence is defined as follows:

(1) $$Confidence=p_{r}\left(Object\right)\times IoU_{pred}^{truth},p_{r}\left(Object\right)\in \{0,1\},$$

where IoU as a standard indicator in target detection defining the detection accuracy by calculating the overlap ration between the true bounding box and the bounding box predicted using detection methods.

If the target falls in the grid, $p_{r}\left(Object\right)=1$, and 0 for otherwise. Then, a tensor of dimensions is predicted by building a single CNN network:

(2) S×S×(B×5+C),

where S×S is the number of grids and each grid can predict B bounding boxes, and C is the number of the object classes in the model.

YOLOV3 includes a CNN feature extractor named Darknet-53 as the backbone, which is a 53 layered CNN network. Compared with the previous versions, YOLOV3 predicts boxes at three different scales and the tensor dimensions are correspondingly changed as follows:

(3) S×S×(3×(4+1+C)),

The loss function of the YOLOV3 constitutes three parts, including coordinate prediction error, IoU error, and classification error, shown as follows:

(4) $$Loss=\sum_{i=1}^{S^{2}}Err_{coord}+Err_{IoU}+Err_{cls},$$

where $S^{2}$ means the number of grids covered in the input image.

The coordinate prediction error is defined as follows:

(5) $$Err_{coord}=\lambda_{coord}\sum_{i=1}^{S^{2}}\sum_{j=1}^{B}I_{ij}^{obj}\left[{\left(x_{i}-{\hat{x}}_{i}\right)}^{2}+{\left(y_{i}-{\hat{y}}_{i}\right)}^{2}\right]\;+\lambda_{coord}\sum_{i=1}^{S^{2}}\sum_{j=1}^{B}I_{ij}^{obj}\left[{\left(w_{i}-{\hat{w}}_{i}\right)}^{2}+{\left(h_{i}-{\hat{h}}_{i}\right)}^{2}\right]$$

where $\lambda_{coord}$ is the weight of the coordinate prediction error. $I_{ij}^{obj}=1$ if the target falls into the *j*th bounding box in grid *i*, otherwise $I_{ij}^{obj}=0$. $\left(x_{i},y_{i},w_{i},h_{i}\right)$ are true values of a target, and $\left({\hat{x}}_{i},{\hat{y}}_{i},{\hat{w}}_{i},{\hat{h}}_{i}\right)$ are information of predicted bounding box in terms of the center coordinate, height, and width.

The IoU error is defined as follows:

(6) $$Err_{IoU}=\sum_{i=1}^{S^{2}}\sum_{j=1}^{B}I_{ij}^{obj}{\left(C_{i}-{\hat{C}}_{i}\right)}^{2}+\lambda_{noobj}\sum_{i=1}^{S^{2}}\sum_{j=1}^{B}I_{ij}^{obj}{\left(C_{i}-{\hat{C}}_{i}\right)}^{2},$$

where $\lambda_{noobj}$ is the weight of the IoU error, $C_{i}$ is the true confidence, and ${\hat{C}}_{i}$ is the predicted confidence.

The classification error is defined as follows:

(7) $$Err_{cls}={\sum_{i=1}^{S^{2}}\sum_{j=1}^{B}I_{ij}^{obj}\sum_{c\in classes}\left(p_{i}\left(c\right)-{\hat{p}}_{i}\left(c\right)\right)}^{2},$$

where c means the class to which the detected target belongs, $p_{i}\left(c\right)$ is the true probability that the target belonging to class c is in grid *i*, and ${\hat{p}}_{i}\left(c\right)$ is the predicted probability.

### 3.2. DenseNet

The targets should be detected as early as possible, especially while the USV sailing at high speed, which could save sufficient time to plan subsequent operations. This requires the detection method to be sensitive to the targets with long distance, even though the features of targets are not enough at this time. Furthermore, for the complex ocean environment, the targets are usually blurred in cases of the foggy weather or the water droplets adhere to the camera lens. These bring a huge challenge in target detection. Although the YOLOV3 is sensitive to the small-scale objects, the features information of the targets is lost in the transmission of the neural network owing to convolution and down-sampling. Therefore, in this paper, DenseNet is proposed to improve the original YOLOV3, which could make more effective use of feature information [31].

**Figure 3:**
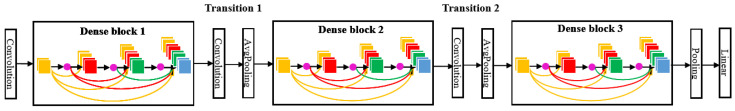
Demonstration of DenseNet structure.

Between the transition layers of the YOLOV3, a structure referred to as the Dense Block is added to ensure all the feature information has no loss in the transition. The structure of DenseNet is demonstrated in Figure 3. The Dense Blocks facilitate feature reuse and mitigate gradient vanishing.

### 3.3. Proposed Method

This paper takes Darknet-53 of YOLO-V3 as the basic network structure for feature extraction. Considering that DenseNet has the characteristics of feature reuse and enhances the feature propagation, the down-sampling layers in Darknet-53 are replaced with DenseNet, which is likely to cause the feature loss.

**Figure 4:**
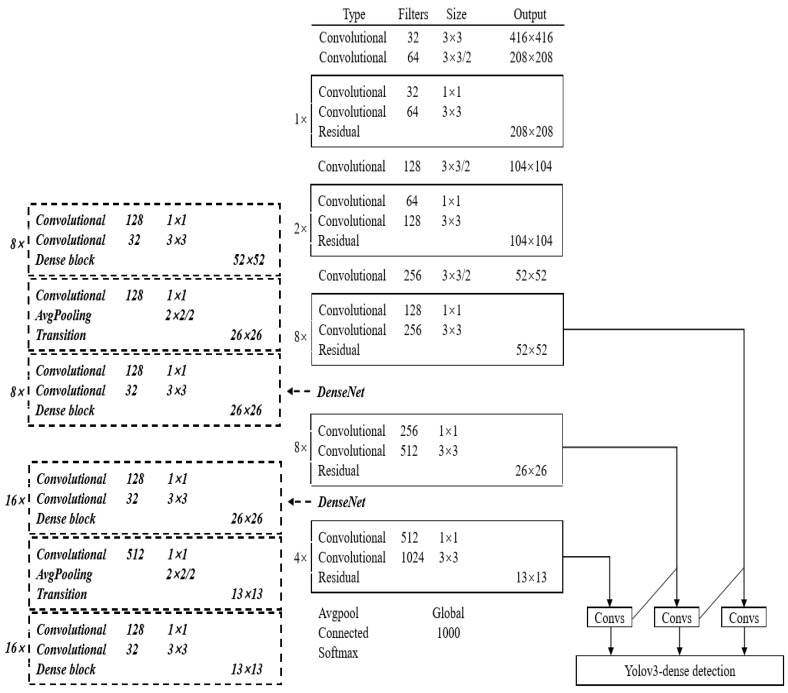
Network structure diagram of YOLOV3-dense.

The network structure diagram of YOLOV3-dense is shown in Figure 4. Considering both the calculating cost and the network structure, the size of the input images is changed from 1280 × 720 to 416 × 416. In the improved YOLOV3 network, the DenseNet structure replaces the 26 × 26 and 13 × 13 down-sampling layers; it contains the dense-block and transition-layer. The transfer function of dense-block is made up of batch normalization (BN), rectified linear units (ReLU), and convolution (Conv), which is used for nonlinear transformation between $x_{0},x_{1},\ldots ,x_{l-1}$ layers. The specific operation is as follows. In the layers with 26 × 26 resolution, the input layer $x_{0}$ first applies BN-ReLU-Conv(1 × 1) operation, and then applies BN-ReLU-Conv(3 × 3) operation and outputs $x_{1}$. $x_{0}$ is spliced with $x_{1}$ as the new input $\left[x_{0},x_{1}\right]$, and the above operation is repeated to output $x_{2}$. $\left[x_{0},x_{1}\right]$ is spliced with $x_{2}$ as the new input $\left[x_{0},x_{1},x_{2}\right]$, and so on. Finally, the feature layer is spliced into 26 × 26 × 512 and propagates forward. In the layers with 13 × 13 resolution, the feature layer finally is spliced into 13 × 13 × 1024 and propagates forward. The transition-layer is used to connect dense-block and the feature map applies BN-ReLU-Conv(1 × 1)-average pooling in this layer to reduce the size.

In the prediction process, the YOLOV3-dense model proposed in this paper predicts the bounding boxes at three different scales: 52 × 52, 26 × 26 and 13 × 13, and improved the detection accuracy of small targets.

## 4. Performance Metrics

### 4.1. Precision, Recall, and F-Measure

To evaluate the detection performance of the proposed YOLOV3-dense model, the original YOLOV3 and the Faster-RCNN-resnet101 are also applied to the detection of targets from realistic images obtained in the sea trial via USV, as well as the YOLOV3-dense model. The precision and recall analysis is utilized as an evaluation method after the detection of targets [32]. Precision refers to the percentage of correctly identified targets from the total extracted results. A high precision value indicates that the detection results contain a high percentage of useful information (true positive, TP) and a low percentage of false alarms (false positive, FP). The false-positive rate discussed in this study refers to the percentage of the false alarms in the total results, which has a value equal to 1 – precision:

(8) $$Precision=\frac{TP}{TP\;+\;FP}.$$

The term recall indicates the accuracy of detecting the target objects (i.e., ships) and refers to the true-positive rate. A high recall value indicates that most of the targets have been detected. The sum of true positive and false negative (FN) equals the actual number of targets in the total images:

(9) $$Recall=\frac{TP}{TP\;+\;FN}.$$

The average precision (AP) can be calculated by the precision–recall curve as follows:

(10) $$AP=\int_{0}^{1}precision(recall)drecall.$$

In the F-measure, both precision and recall are taken into account to evaluate the overall performance of object detection. A high F-measure score indicates that the detection results contain fewer false alarms and more correct detections. The F-measure is calculated as follows:

(11) $$F-measure=\frac{2\times Precision\times Recall}{Precision\;+\;Recall}.$$

### 4.2. Average Detection Time Cost

Otherwise, the average detection time cost is also evaluated in the experiments for the reason that the time cost is related to the feasibility of real-time application in practice.

## 5. Experimental Results and Discussions

Some experiments were implemented to evaluate the performance of the proposed model. A total of 3000 original image datasets were carried out in these experiments, which were randomly subdivided into three groups including the training dataset, validation dataset, and testing dataset. The completed dataset distribution in experiments is shown in Table 3, and all experiments are run on a server equipped with Intel XEON Gold 5217 CPU and NVIDIA RTX TITAN GPU cards.

### 5.1. Detection Performance Evaluation

**Figure 5:**
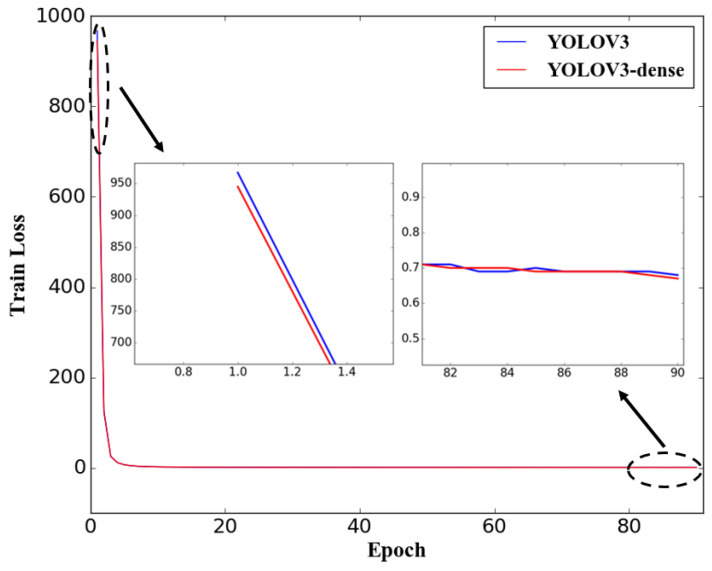
Loss curves of YOLOV3-dense, YOLOV3, and faster region-based convolutional neural network (R-CNN).

The loss curves of the proposed YOLOV3-dense and the YOLOV3 during 45 thousand iterations are shown as Figure 5. The loss of both the two models decreases gradually with the increase of the iteration, and eventually converges to a low constant. After the 45 thousand iterations, the final loss of the proposed YOLOV3-dense is 0.67, while the final loss of the original YOLOV3 is 0.68. It is notable that the proposed YOLOV3-dense has a slightly higher convergence speed compared with YOLOV3 in the early stages of training, which means the weights of the proposed method could be trained with a lower time cost.

**Figure 6:**
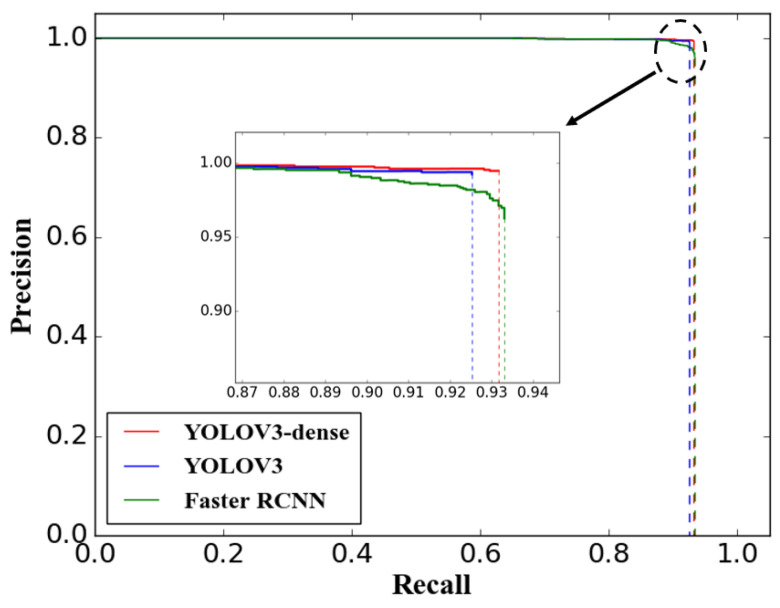
Precision–recall curves of YOLOV3-dense, YOLOV3, and Faster R-CNN.

The evaluation index covering TP, FP, AP, and F-measure of the proposed YOLOV3-dense model and the other comparison models are listed in Table 4, and the precision–recall curves for these models during testing are shown in Figure 6.

On the basis of the above results, the F-measure of the proposed YOLOV3-dense model is 0.962, which is higher than the other two models. This indicates that the comprehensive performance of the YOLOV3-dense balancing the performance of both precision and recall is superior to the other two models. The AP of YOLOV3-dense is higher than YOLOV3 and basically equal to Faster R-CNN. The YOLOV3-dense predicted 1317 targets in the testing image data with 1406 ground truth. The performance of YOLOV3-dense in the index TP and FP is better than YOLOV3. The Faster R-CNN predicted 1363 targets, which is more than both YOLOV3-dense and YOLOV3. The TP of Faster RCNN increases only two times compared with YOLOV3-dense, but the FP of Faster R-CNN reaches 51, more than 7 times higher than YOLOV3-dense. This indicates that the Faster R-CNN has a higher false alarm compared with YOLOV3-dense; in other words, more noises are falsely identified as targets when Faster R-CNN is implemented. The experimental results demonstrated that the overall detection performance of the proposed YOLOV3-dense is superior to the other two models.

### 5.2. Real-Time Performance Evaluation

The average detection time cost of the proposed YOLOV3-dense model is 67.5 ms for one testing image data, which is 10 ms slower than YOLOV3 because more features were processed in the YOLOV3-dense model. However, this detection speed of YOLOV3-dense is enough for the applications with USV in real time. It notable that the average detection time cost of Faster R-CNN is 963.8 ms, more than 14 times slower than the YOLOV3-dense model, though the detection performance of Faster R-CNN is slightly better than YOLOV3-dense. However, this is difficult to apply on the USV to detect the targets, especially for the fast-moving targets.

### 5.3. Performance of Data Augmentation

**Figure 7:**
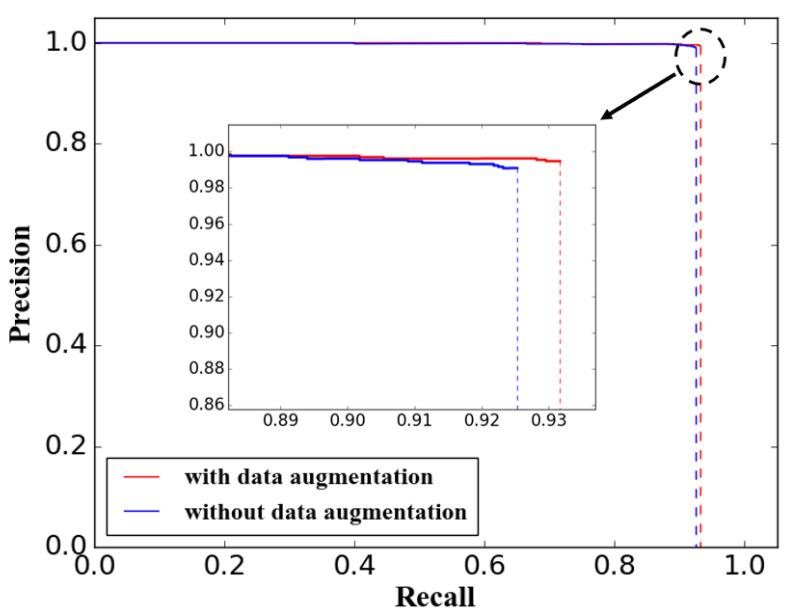
Precision–recall curves of YOLOV3-dense models to evaluate the performance of data augmentation.

Brightness and rotation transform were used to augmented the training data to simulate the changes of the light and the ocean environment. For the purpose of evaluating the influence of the data augmentation to target detection performance, 1000 original image data and 4000 augmented image data are utilized as input to train the proposed YOLOV3-dense model, respectively. The components of the augmented data are the same as those shown in Table 2, and the same 800 testing image data were utilized to evaluate the performance. The results are shown in Table 5 and the precision–recall curves for these two models are shown in Figure 7.

The AP and F-measure of the model trained without the data augmentation are 92.44% and 0.957, respectively, while these evaluation indexes of the model trained with the data augmentation are 93.13% and 0.962, respectively. The AP and F-measure are increased by 0.69% and 0.005, respectively, through the operation of data augmentation, which verified that the data augmentation is to some extent effective to improve the detection.

### 5.4. Performance under Different Environment Conditions

**Figure 8:**
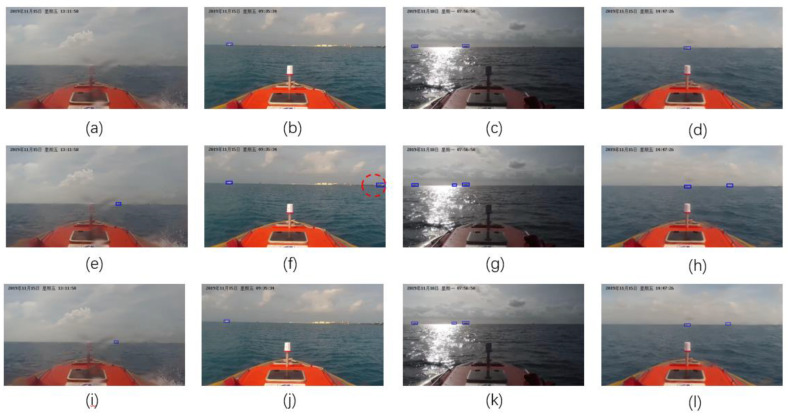
Detection results under different envrionment conditions. YOLOV3: (**a**–**d**); Faster R-CNN: (**e**–**h**); YOLOV3-dense: (**i**–**l**).

In the realistic environment, the changes of the light and weather, as well as the water droplets adhering to the lens of the camera, would influence the target detection performance. All the detection results were reviewed manually, and the typical detection results under different environmental conditions are illustrated in Figure 8.

The upper, middle, and lower row of Figure 8 listed the detection results achieved under different environmental conditions by implementing the model YOLOV3, Faster R-CNN, and proposed YOLOV3-dense, respectively. In the case of the scattering of light caused by water droplets adhering to the lens of the camera (Figure 8a,e,i) the YOLOV3-dense and Faster R-CNN can properly identify the target falling into the region of water droplets. For the case of light reflection (Figure 8c,g,k) and the case of cloudy weather (Figure 8d,h), and Figure 8l), the YOLOV3-dense and Faster R-CNN can also properly identify more targets than YOLOV3. These validate that DenseNet is conducive to improving the detection performance in different environmental conditions. Otherwise, the proposed YOLOV3-dense has the effect of suppressing the false alarm (marked with a red dotted circle in Figure 8f) compared with the Faster R-CNN. These experimental results further demonstrate that the proposed YOLOV3-dense in this paper is robust against the changes in the environmental conditions.

## 6. Conclusions

This study proposed an improved YOLOV3 model by fusing DenseNet to detect sea surface targets under different environmental conditions, which is expected to enhance the environmental adaptability of the USV during a long-term task. The YOLOV3-dense model proposed in this paper takes advantage of DenseNet’s feature reuse character to optimize the sample layer of the feature extraction part in the YOLOV3 model, and to promote feature propagation. The realistic images obtained in the sea trial via USV are used to train models and evaluate the performances of the proposed YOLOV3-dense model compared with the model YOLOV3 and Faster R-CNN with ResNet-101. The F-measure of the proposed YOLOV3-dense model is 0.962, which is higher than YOLOV3 (0.958) and Faster R-CNN (0.954). Simultaneously, the AP of YOLOV3-dense achieves 93.13%, which is higher than YOLOV3 (92.47%) and basically equal to Faster R-CNN (93.21%). However, the Faster R-CNN has a higher false alarm compared with YOLOV3-dense, as the TP of Faster R-CNN is much higher. These experimental results show that the YOLOV3-dense model proposed in this paper is superior to the YOLOV3 model and has better overall performance compared with the Faster R-CNN with ResNet-101. Besides, the YOLOV3-dense model is robust to the weather changes of the realistic ocean environment and meets the requirement of the real-time prediction (67.5 ms/frame) for USVs.

The focus of future work will be on deploying the proposed model as a hardware module on USVs and implementing it to detect sea-surface targets in actual tasks. Moreover, the detection model will be optimized to accelerate the training process and further improve the detection performance.

## Figures and Tables

**Table 1:** Environmental conditions and basic parameters of the collected images.

Condition	Size	Number	Total Number
Sunny	1280 × 720	1356	3000
Cloudy		696	
Sea flog		861	
Water droplets adhere to lens		87	

**Table 2:** Components of training dataset.

	Original Data	Brightness	Rotation	Total
Training Dataset	1000	1000	2000	4000

**Table 3:** Components of experiment dataset.

	Original Data	Augmented Data	Total
Training Dataset	1000	3000	4000
Validation Dataset	1200	NA	1200
Testing Dataset	800		800

**Table 4:** Detection performance for these models. TP, true positive; FP, false positive; AP, average precision; R-CNN, region-based convolutional neural network.

Model	Ground-Truth	Predicted	TP	FP	AP	*F*-Measure
YOLOV3	1406	1311	1301	10	92.47%	0.958
Faster RCNN		1363	1312	51	93.21%	0.954
YOLOV3-dense		1317	1310	7	93.13%	0.962

**Table 5:** Performance of data augmentation.

Model	Iteration	AP	*F*-Measure
Training data without data augmentation	45,000	92.44%	0.957
Training data with data augmentation		93.13%	0.962
